# Antimicrobial prescribing in long-term care facilities: a nationwide point-prevalence study, Slovenia, 2016

**DOI:** 10.2807/1560-7917.ES.2018.23.46.1800100

**Published:** 2018-11-15

**Authors:** Dora Stepan, Lea Ušaj, Marija Petek Šter, Marjetka Smolinger Galun, Hermina Smole, Bojana Beović

**Affiliations:** 1 Department of Infectious Diseases, Faculty of Medicine, University of Ljubljana, Ljubljana, Slovenia; 2 Department of Family Medicine, Faculty of Medicine, University of Ljubljana, Ljubljana, Slovenia; 3 Long-term care facility Danice Vogrinec Maribor, Maribor, Slovenia; 4 Long-term care facility for the elderly Trebnje, Trebnje, Slovenia; 5 Department of Infectious Diseases, University Medical Centre Ljubljana, Ljubljana, Slovenia

**Keywords:** antibiotics, long-term care facility, multidrug-resistant bacteria, antibiotic prescribing, antibiotics stewardship

## Abstract

Residents in long-term care are at high risk of infections because of their old age and many related health problems that lead to frequent antibiotic prescribing. The aim of the study was to assess antibiotic use in Slovenian long-term care facilities (LTCFs). The point-prevalence study was conducted between April and June 2016. Online questionnaires were sent to all Slovenian LTCFs. Eighty (68.4%) of the 117 LTCFs contacted, caring for 13,032 residents (70.6% of all Slovenian LTCF residents), responded to the survey. On the day of the study, the mean antibiotic prevalence per LTCF was 2.4% (95% confidence interval: 1.94–2.66). Most (70.2%) of the residents taking antibiotics were female. Most residents were being treated for respiratory tract (42.7%) or urinary tract (33.3%) infections. Co-amoxiclav and fluoroquinolones were the most frequently prescribed antibiotics (41.0% and 22.3% respectively). Microbiological tests were performed for 5.2% of residents receiving antibiotics. Forty nine (19.8%) residents receiving antibiotics were colonised with multidrug-resistant bacteria (MDR). Antibiotic use in Slovenian LTCFs is not very high, but most prescribed antibiotics are broad-spectrum. Together with low use of microbiological testing and high prevalence of colonisation with MDR bacteria the situation is worrisome and warrants the introduction of antimicrobial stewardship interventions.

## Introduction

All European Union countries have seen an increase in the population aged 65 years and over in the past 10 years. In many countries, including Slovenia, elderly people represent one fifth or more of the population [1]. In Organisation for Economic Co-operation and Development member countries in 2011, the number of long-term care beds ranged from 17.5 to 81.7 per 1,000 inhabitants 65 years and older [2]. Residents in long-term care are at high risk of infections because of their old age and age-related health problems that lead to frequent antibiotic prescribing. A systematic literature review showed that 47% to 79% of long-term care facility (LTCF) residents receive antibiotics each year [3]. In addition, LTCFs may represent foci for multidrug-resistant bacteria [4].

Antimicrobial stewardship interventions in nursing homes are needed to provide effective treatment for patients with infection and avoid excessive and inappropriate use that may aggravate antimicrobial resistance in the facilities and beyond [5]. The first step towards improved antimicrobial prescribing is to analyse the current patterns of antimicrobial use. Several studies on antimicrobial use in LTCFs have been published in the past few years, but with some exceptions [6-10], the studies included relatively few LTCFs from one country. Slovenia was included in the Healthcare-Associated Infections in Long-Term Care Facilities Project (HALT) in 2010 with six LTCFs and in the 2013 HALT-2 study with four LTCFs; in the latter study the Slovenian LTCF sample was not representative [11,12]. The aim of this study was to provide a deeper insight into antibiotic prescribing patterns in Slovenian LTCFs.

## Material and methods

### Study population

In Slovenia, there are 129 LTCFs, 12 of which are specialised nursing homes for adults; all other LTCFs serve mixed populations [13]. After excluding specialised institutions, we invited 117 Slovenian LTCFs, comprising 18,457 residents, to take part in our study. The contact information for all LTCFs in the country was obtained from the website of the Association of Social Institutions in Slovenia [13]. Data on age and sex of residents were obtained from the Association’s 2016 report [14]. All residents who were receiving systemic antibiotic treatment and who gave consent to the study were included in the analysis.

### Study design and time schedule

For our point-prevalence study, we used an adapted version of the HALT protocol [12]. The study was conducted in each facility in the time window between 1 April and 30 June 2016. On the day of the study, data on patients on antimicrobial treatment and the facility were collected simultaneously. The directors, chief nurses and medical doctors of each LCTF were informed about the survey in advance, but the exact day of the survey was communicated to the LTCF only 1 or 2 days before the survey day.

### Data collection

Data were collected either by an LTCF employee (most often a (head) nurse) or, in the case of larger facilities, a local researcher supported by the survey coordinators. On the day of the study, the study coordinators were in contact by phone or in person with all local researchers, who collected the data themselves to ensure the correct execution of the survey. All facilities were asked to fill in two online questionnaires. The first was an institutional questionnaire on LTCF characteristics and population (numbers of residents, wheelchair users, bedridden residents, residents with dementia, residents taking antimicrobial treatment and characteristics of the physician working in the facility). The second was a questionnaire for each resident receiving systemic antibiotic treatment on the day of the study. It explored the resident’s characteristics (age, sex), antimicrobial use (compound name, indication for therapy, prescribed doses, route of administration), risk factors (presence of urinary catheter, vascular catheter and wounds), care-load indicators (faecal and/or urinary incontinence, dementia, impaired mobility). We asked who prescribed the antibiotic treatment and which diagnostic tests were performed to diagnose infection. Colonisation with multidrug-resistant bacteria (meticillin-resistant *Staphylococcus aureus* (MRSA), vancomycin-resistant *Enterococcus* (VRE), extended-spectrum beta-lactamase (ESBL) producing enterobacteria, carbapenem-resistant Enterobacteriaceae (CRE)) in patients receiving antibiotics was recorded from the residents’ documents. We included all oral, intramuscular and intravenous systemic antibiotic treatments. Topical antibiotics, antivirals, antifungals and antiseptics were excluded, as well as mupirocin nasal ointment for MRSA decolonisation.

### Data analysis

Numeric variables were presented with arithmetic mean (x), median, range (highest and lowest value) and standard deviation (sd). Descriptive variables were presented as rates and percentages. Statistical significance was assessed with the chi-squared test and odds ratios were calculated with R 3.3.1 (The R Foundation for Statistical Computing, Vienna, Austria).

### Ethical considerations and confidentiality

The study was approved by National Medical Ethics Committee of the Republic of Slovenia (n. 0120–568/2015–4, KME 32/12/15). Informed consent to collect relevant data was obtained from residents or, when residents were considered by nursing staff to lack the capacity to consent, their next of kin. To ensure confidentiality, the residents’ data were anonymised and unique LTCF and resident numbers were recorded in the questionnaires. The link between the labels given to the LTCF and the patients was discarded after data analysis.

## Results

Eighty out of 117 Slovenian LTCFs (68.4%) responded to our invitation, and 13,032 (70.6%) residents participated in our survey. On the day the survey was conducted, 317 of 13,032 residents received antibiotics (2.4%; median: 1.9%; range: 0–7.6%; 95% confidence intervals (CI): 1.94–2.66%). Further analysis of the per-patient data was performed on the population of 255 patients (2.0% of residents in the LTCFs included in the study) who gave informed consent. Some responses were missing for up to 3% (8/255) of residents in the study. The characteristics of the participating LTCFs are presented in detail in Table 1.

**Table 1:** Characteristics of the facilities and residents included in the study on antimicrobial prescribing in long-term care facilities, Slovenia 2016 (n = 80 facilities)

Variable	n	%
Number of residents in participating LTCFs	13,032	100
Mean number of residents per facility	163 (range 21–608)	NA
Number of wheelchair users	3,693	28.3
Number of bedridden residents	3,511	26.9
Number of residents with dementia	5,467	42.0

LTCF: long-term care facility; NA: not applicable.

The majority of the physicians (80%; 80/100) who prescribed the antibiotic treatment worked in other institutions beside the LTCF (health centre, hospital), the remaining 20% (20/100) worked only in the facility. The antibiotic treatment for 208 of 247 residents (84.2%) was prescribed by general practitioners working in the LTCF; for 17 cases (6.9%) the treatment was started in hospital, for 11 cases (4.5%) the antibiotics were prescribed in specialist clinics, and four antibiotic therapies (1.6%) were prescribed by a doctor on duty.

The mean age of the residents with an antimicrobial treatment was 83.4 years (median: 85 years; range: 46–100 years), 179 (70.2%) were female. Other characteristics of the residents included in the study are presented in Table 2.

**Table 2:** Characteristics of the residents receiving antibiotics and included in the analysis, study on antimicrobial prescribing in long-term care facilities, Slovenia 2016 (n = 255)

Characteristic	Total		Male		Female	
	n	%	n	%	n	%
**Indication for antibiotic treatment**	255^a^	100^a^	76^a^	100^a^	179^a^	100^a^
Respiratory tract infection	109	42.7	31	40.8	78	43.6
Urinary tract infection	85	33.3	24	31.6	61	34.1
Skin and skin structure infections	50	19.6	17	22.4	33	18.4
Gastrointestinal infections	3	1.2	1	1.3	2	1.1
Prophylaxis	3	1.2	1	1.3	2	1.1
Other	12	4.7	4	5.3	8	4.5
**Associated diseases and risk factors for various infections**	247^a,b^	100^a^	73^a^	100^a^	174^a^	100^a^
Urinary catheter	34	13.8	18	24.7	16	9.2
Vascular catheter	2	0.8	0	0	2	1.1
Urinary incontinence	189	76.5	46	63.0	143	82.2
Faecal incontinence	148	60.0	44	60.3	104	59.8
Wounds, ulcers	53	21.5	21	28.8	32	13.4
Dementia	99	40.1	28	38.4	71	40.8
Wheelchair-users	77	31.2	22	30.1	55	31.6
Bedridden residents	95	38.5	28	38.4	67	38.5
Other (post cerebrovascular insult, nasogastric tube etc.)	23	9.3	10	13.7	13	7.5
No risk factor	18	7.3	6	8.2	12	7.0

^a^ More than one category possible in one individual.

^b^ Information available for 247 residents.

Detailed data on antibiotics were available for 251 residents, seven residents (2.8%) received two antibiotic agents simultaneously. 241 residents (96.0%) received antimicrobial treatment per os, only two residents received parenteral antibiotic treatment (co-amoxiclav intravenously or gentamicin intramuscularly), seven residents (2.8%) received treatment per nasogastric tube (co-amoxiclav, cefixime, ciprofloxacin, moxifloxacin) and one per percutaneous gastric tube (co-amoxiclav). Co-amoxiclav was the most frequently prescribed antibiotic overall, used in 14.1% of urinary tract infections (UTI), 61.7% of respiratory tract infections (RTI) and 48.7.0% of skin and skin structure infections. Fluoroquinolones were the second most commonly prescribed antibiotics, with ciprofloxacin being the most common in this group; ciprofloxacin was prescribed in 31.8. % of UTI cases (Supplement[Fig f1]). Detailed information on the antibiotic treatments by indication is presented in the Supplement.

**Figure f1:**
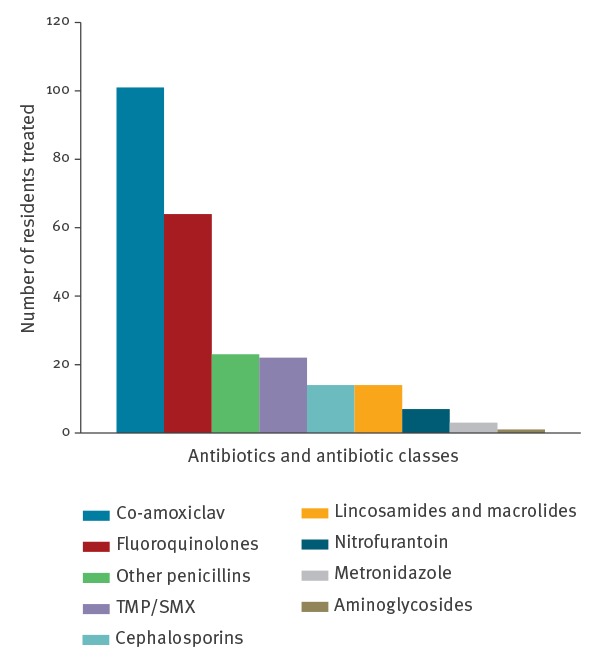
Distribution of prescribed antibiotics and antibiotic classes, study on antimicrobial prescribing in long-term care facilities, Slovenia, 2016 (n = 251)

The commonest diagnostic tests used in RTI were C-reactive protein (66/105, 62.9%) and blood cell count (in 60/105, 57.1% of RTI), the urine dipstick test was performed in 71/83 (85.5%) of UTI cases, whereas for most skin infections (28/48, 58.3%) no diagnostic tests were done. Microbiological testing was performed in 13/245 (5.3%) of cases.

Forty nine (19.8%) of 247 residents receiving antibiotics were colonised with multidrug-resistant bacteria. Specifically, 39/247 (15.8%) of residents receiving antibiotics were colonised with ESBL-producing bacteria, 11/247 (4.5%) with MRSA and there were two cases of CRE. No cases of VRE colonisation were found. Three residents were colonised with multiple multi-drug resistant micro-organisms (MDRO), of whom two had MRSA and ESBL and one was colonised with ESBL and CRE. Of the 36 residents colonised only with ESBL-producing bacteria, most received co-amoxiclav (n = 9), followed by fluoroquinolones (n = 6) and TMP/SMX (n = 5). Of 11 residents colonised with MRSA, four residents received co-amoxiclav.

### Risk factors for antimicrobial use

According to the Slovenian statistics office, 63% of LTCF residents in 2016 were 80 years old and older [14]. In our study, the share of residents receiving antibiotics who were ≥ 80 years old was 72.9%, the difference was statistically significant (p < 0.01, chi-squared test, OR = 0.626). Our study had 9,005 female residents who participated (69.1%) and 4,027 male residents (30.9%). Of these, 179 women and 76 men received antibiotic treatment. Sex was not significantly correlated with antibiotic prescribing (p = 0.702, chi-squared test, OR = 1.054). Dementia and being wheelchair-user were not significantly correlated with antibiotic prescribing (p = 0.307, chi-squared test, OR = 0.876, and p = 0.506, chi-squared test, OR = 1.096 respectively), however antibiotics were prescribed more often for bedridden residents (p < 0.01, chi-squared test, OR = 1.627) (Tables 1 and 2).

## Discussion

This is the first nationwide study to investigate antibiotic prescribing in LTCFs in Slovenia. Of the 13,032 residents included in the study, 317 residents (2.4%) received antimicrobial treatment on the day of our survey. Several other studies have investigated the prevalence of antibiotic use in LTCFs with similar methodology. European Surveillance of Antimicrobial Consumption (ESAC) conducted research in 21 European countries in April 2009 that included 323 LTCFs; the mean prevalence of antimicrobial treatment was 6.3% with a range from 1% to 17.3%. Slovenia was also included in the survey, with six LTCFs, and the antibiotic prevalence was 3.59% [15]. Another report from the same ESAC project included 85 LTCFs from 15 countries in April and in November 2009; the mean antibiotic prevalence was 6.5% and 5.0% respectively [16]. Two point-prevalence surveys supported by the European Centre for Disease Prevention and Control (ECDC) were performed in 2010 and 2013 [11,12]. Both surveys reported on the varying usage of antimicrobials in different European countries, including Slovenia. In HALT, the mean prevalence of antimicrobial treatment was 4.3% (range: 0.0–13.3%). Slovenia was represented with six LTCFs, and the prevalence of antibiotic treatment was 2.3% [11]. In the HALT-2 study, the mean European prevalence of antimicrobial treatment was 4.4% (range: 1–12.1%) [12]. Slovenia was also included in HALT-2, but because there were only two participating LTCFs, the results were poorly representative. A point-prevalence study was also carried out in 44 Norwegian nursing homes in spring 2006. Of the 1,473 nursing home residents, 224 (15%) were prescribed antibiotics [17]. Our survey showed lower antimicrobial use in Slovenian LTCFs than in several other European countries. The results are in line with the data on the consumption of antimicrobials for systemic use in the community (primary care sector) in Europe from 2016. Slovenia was the country with the sixth lowest prevalence of antimicrobial prescriptions (13.9 defined daily doses (DDD)/1,000 inhabitants/day) [14].

However, the prevalence of antibiotic use in LTCFs may not be comparable because of the different types of LTCFs included in the studies [18]. In our study we included mixed LTCFs, which were also the main types of LTCFs included in the HALT studies [11,12]. The mean age of residents on antimicrobial treatment in our study was 83.4 years, which is only slightly higher than the mean age in the HALT (82.5 years) and in HALT-2 (81.8 years) studies and comparable to the two ESAC reports (83 years). The population in the Norwegian study was older than in Slovenian LTCFs (76% vs 66.8% of residents aged 80 years or older) and some of the residents lived in facilities which specialised in dementia care [17,19,20].

When comparing surveys, we must also consider differences in the data collection time [18]. Our study was conducted between April and June 2016 when the influenza season was over. The differences in the time period of evaluation and the particular meteorological details of the years studied also might explain the differences between the percentages of antibiotic use in our study and the above-mentioned surveys.

In our study we found that residents receiving antibiotic treatment were older than the LTCF population in general. A Finnish study which analysed antibiotic treatments in LTCFs over a 1-month period found age below 85 years to be a risk factor for antibiotic therapy [19]. Most residents receiving antibiotics in our study were female (70%), which is similar to the findings of all previously mentioned studies [11,12,15-17]. Surprisingly, in a Canadian study which analysed antibiotic prescribing in LTCFs during a 1-year period, 74% of residents receiving antibiotics were men (74%) [20]. The prevalence of antibiotic treatments in female residents in our study reflects the predominance of female population among the LTCF residents. No influence of sex on the prevalence of antibiotic use was found in the HALT study, and there were slightly fewer female residents receiving antibiotics in comparison with the general LTCF population in HALT-2 [12]. The Finnish study found female sex to be a risk factor for antibiotic therapy [19].

Prophylaxis was given to only 1.2% of residents in our study, which that is much less than other European studies: in the HALT and HALT-2 studies, prophylaxis was given to 27.7% and 27.2% of residents receiving antibiotics, and in the Norwegian study, prophylactic use was even more frequent than therapeutic use [11,12,17]. Low prophylactic use seems to be a Slovenian speciality, since it has already been reported in the HALT and HALT-2 studies, but the difference may be partly explained by our questionnaire where prophylaxis was not specified by anatomical site, and antibiotics given as prophylaxis for UTIs were could possibly be marked under the ‘UTI’ box and not the ‘prophylaxis’ box.

In our study antibiotics were most commonly prescribed for RTIs followed by UTI use. In the Finnish, Norwegian and Swedish studies, UTI use outnumber the RTI use, and in the European international studies, the relative frequency of indication varied from country to country with the predominance of RTIs or UTIs [11,12,17,19,21]. We may assume that the differences do not only reflect different incidence of infections but also the diagnostic approach of physicians.

Penicillins were the most commonly prescribed antibiotic class in our survey, and also in the two European studies [11,12]. More worrying is the high use of co-amoxiclav, which was prescribed far more often than other penicillins (Figure). Another problematic finding is the high prevalence of fluoroquinolones. Co-amoxiclav and fluoroquinolones are broad-spectrum antibiotics which have been linked to side-effects including *Clostridium difficile* infections and antimicrobial resistance [22-24]. The same pattern of co-amoxiclav followed by fluoroquinolones as the most commonly prescribed antibiotics was found in a French study [10]. In the HALT and HALT-2 studies, most patients received penicillins variously co-prescribed with co-amoxiclav, other antibacterials (J01X, mostly nitrofurantoin) and fluoroquinolones. In the contrast, in Norway most residents received therapy with pivmecillinam or penicillin V [11,12,17]. In our survey most residents received oral treatment. In the HALT and HALT-2 study oral administration of antibiotics was most common, but in some countries such as Italy, Bulgaria and Spain, a large proportion of antibiotics were given parenterally [11,12]. Most antibiotics in our survey and in several other studies including the two European surveys [11,12] were prescribed by primary care physicians or doctors working in the facilities, which gives an opportunity for efficient educational and other antimicrobial stewardship interventions.

Our study has several limitations. We were not able to include all LTCFs in the country, and we chose a simplified approach compared to the ECDC HALT protocols due to limited resources [12]. We did not collect microbiology results, we only collected the number of tests done. We did not classify the facilities, but excluded specialised facilities as described above. There was no strict case definition, diagnosis of the infections was obtained from patient records. Since we required informed consent from every patient (or their family) on antibiotics if we wanted to collect patient-related data, we were unable to collect detailed data on residents receiving antibiotics who did not sign informed consent, or to perform detailed analysis of patient data for the whole cohort of patients on antibiotics. In addition, we did not check the appropriateness of antibiotic therapy. Colonisation was only recorded in residents receiving antibiotics and not in other LTCF residents, and it was only derived from the medical records, not microbiological testing. Consequently, we were not able to draw any additional conclusions important for the potential interventions. We were only able to compare the sex and the age of the residents receiving antibiotics with the data from the literature that limits the relevance of statistical comparison. However, the study gives the first complete insight into antibiotic prescribing in LTCFs in Slovenia, which is needed for any further antimicrobial stewardship activity in the country.

Dementia was diagnosed in 40% of patients receiving antibiotics in our study, but in contrast with some other studies [25,26] a dementia diagnosis among residents receiving antibiotics was not more common than in other residents. In the Finnish study, antibiotic therapy was more common in patients with reported confusion [19]. Immobility was not associated with higher antibiotic use in wheelchair users, significant association was only found for bedridden residents. Being bedridden was identified as risk factor for antibiotics also by the Finnish authors [19].

Almost one fifth (19.2%) of residents receiving antibiotics in our study were colonised with multidrug-resistant microorganisms, and most of them harboured ESBL-producing bacteria. High colonisation rates were found in other studies [27,28], but different methodologies prevent the comparison of our data. We have not investigated the causative agents of infections in the residents receiving antibiotics, but the mismatch between the susceptibility of the colonising bacteria and prescribed antibiotics points to potentially ineffective antibiotic therapy in at least some cases. The use of microbiology tests in the study population (in only 5.2% of cases), is much lower than reported in the HALT studies, and increases the possibility of under-treatment, despite the fact that patients receiving treatment are generally prescribed broad-spectrum antibiotics such as co-amoxiclav and fluoroquinolones [11,12].

In conclusion we may say that the use of antibiotics in Slovenian LTCFs is not high. More problematic is the frequent use of co-amoxiclav and fluoroquinolones, broad-spectrum antibiotics known as drivers of resistance, and the cause of several important side effects. Almost exclusive empirical antibiotic use and an already-high colonisation rate with multidrug-resistant bacteria give an impression of potentially inappropriate and ineffective antibiotic treatment. Introduction of antimicrobial stewardship including guidelines for diagnostics and therapy of infections in fragile elderly population in Slovenian LTCFs should be a priority. Special attention should be paid to the most vulnerable bedridden residents.
